# The Effect of *Calendula officinalis* on Oxidative Stress and Bone Loss in Experimental Periodontitis

**DOI:** 10.3389/fphys.2017.00440

**Published:** 2017-06-28

**Authors:** Mariana dos Reis Lima, Amanda P. Lopes, Conceição Martins, Gerly A. C. Brito, Virgínia C. Carneiro, Paula Goes

**Affiliations:** ^1^Nucleus of Study and Research in Pain, Inflammation, and Osteoimmunology, Department of Morphology, Medical School, Federal University of CearáFortaleza, Brazil; ^2^Nucleus of Study and Research in Pain, Inflammation, and Osteoimmunology, Department of Pathology and Legal Medicine, Medical School, Federal University of CearáFortaleza, Brazil; ^3^Department of Morphology, Medical School, Federal University of CearáFortaleza, Brazil

**Keywords:** *Calendula officinalis*, periodontitis, bone loss, oxidative stress, rats

## Abstract

Periodontitis is associated with reduced antioxidant capacity and increased oxidative damage. Oxidative stress induces inflammation and bone loss contributing to the pathological progression of periodontal disease. *Calendula officinalis* (CLO) has demonstrated anti-inflammatory and anti-oxidant activities. Therefore, the aim of this study was to evaluate the effect of CLO on oxidative stress and bone loss in rats subjected to experimental periodontitis (EP). For this, 72 male Wistar rats were divided into groups: Naïve, Saline (SAL) and CLO. Rats received SAL or CLO (90 mg/kg) 30 min before ligature and daily until the 11th day. Naïve group experienced no manipulation. After 11 days, the animals were euthanized and left maxillae collected for macroscopic analysis of alveolar bone loss (ABL). Periodontium was analyzed by macroscopy, scanning electron microscopy; confocal and light polarized microscopy. Immunohistochemical examination of DKK1, WNT 10b and β-catenin was performed. The gingival tissue was collected to reduced glutathione (GSH), superoxide dismutase (SOD), catalase (CAT) and malondialdehyde (MDA) analyses. The 11 days of ligature induced bone loss, breakdown of collagen fibers, increased the immunostaining DKK-1 while reduced WNT 10b and β-catenin expressions. Periodontitis reduced GSH, SOD, CAT and increase MDA. All findings were reversed by 90 mg/kg of CLO. In summary our findings demonstrated that CLO reduced oxidative stress and bone loss and preserved collagen fibers in rats with EP, with participation of WNT signaling pathway.

## Introduction

Periodontitis is a high prevalent infect-inflammatory disease (Petersen and Ogawa, 2005). It is considered the second more important cause of tooth loss on the population (Kayal, 2013). Its etiology is multifactorial, where dental biofilm stimulates immunoinflammatory host response resulting in tissue destruction (Redlich and Smolen, 2012).

During periodontitis, the inflammatory process is marked by neutrophils that invade periodontium and induces the release of proteolytic enzymes and production of reactive oxygen species (ROS) (D'Aiuto et al., 2010). An increased generation of ROS initiates a chain reaction of degradation steps, termed lipid peroxidation, that can ultimately decompose into secondary products such as malondialdehyde (MDA) (Busch and Binder, 2016). In the other hand, the body produces a variety of defense mechanisms to combat the excess of ROS, among these there are the antioxidants (AOs) (Palwankar et al., 2015). Superoxide dismutase (SOD), catalase (CAT) and glutathione (GSH) peroxidase are important AOs that enzymatically eliminate, hydrogen peroxide (H_2_O_2_), oxygen superoxide (O_2_) or the hydroxyl radical (OH), highly reactive oxygen radicals, which are responsible for most oxidative stress in cells (Galli et al., 2011).

The continuous exacerbation of inflammation culminates with collagen fiber destruction and bone resorption (Zheng et al., 2015). Literature has described the role of RANK-RANKL-OPG axis on bone loss (Graves et al., 2011). However, recently another signaling pathway has stood out as a regulator of bone homeostasis, known as the canonical WNT pathway (Kobayashi et al., 2016). Briefly, on bone tissue, WNT proteins, mainly WNT10b, interacts with its receptor (LPR5/6) and induce cytoplasmatic inhibition of GSK3β, which stabilizes β-catenin, which in turn, gain access to the nucleus activating transcriptors factors that promote osteoblast differentiation (Baron and Kneissel, 2013). DKK acts as a regulator of this pathway blocking the interaction between WNT and its receptor, with consequent β-catenin degradation and lack of osteoblast differentiation (Baron and Kneissel, 2013). The expression of WNT pathway inhibitors seems to be induced by inflammatory mediators (Rauner et al., 2008). In this context, knowing that inflammation plays an important role on bone disorders, seems interesting to study the effect of pharmacological agents that may modulate the inflammatory process.

CLO is annual herb well known by its anti-inflammatory activity (Parente et al., 2012). More than 100 constituents have already been identified from the crude extract of this plant, but the flavonoid quercetin is considered of great importance due to its anti-inflammatory and antioxidant effects (Li et al., 2016). It has been reported that CLO extract inhibited significantly the paw edema and inflammation, lowered LPS-induced IL-1, and -6, TNF-alpha, interferon and acute-phase proteins levels (Preethi et al., 2009). In periodontal diseases, as a gel or mouthwash, CLO has shown positive effect on gingivitis treatment (Lauten et al., 2005; Machado, 2010). Specifically in periodontitis, this is the first time that the effect of CLO is studied. Therefore, considering that inflammation and oxidative stress can lead to bone loss, which is the main cause of periodontitis, and that CLO has an important anti-inflammatory and antioxidant effects, it seems interesting to evaluate the effect of CLO in rats subjected to experimental periodontitis.

## Methods

### Animals

Seventy-two male Wistar rats (±200 g) (*Rattus novergicus*), from our own facilities, were used in this study. The animals were kept in cages with temperature-controlled rooms, with food and water *ad libitum* throughout the experiment. In accordance to the Ethical Principles for Animal Research, all efforts were done in order to reduce pain or discomfort to the animals. All procedures and animal treatments were conducted after approval by the institutional Ethical Committee for Animal Research from Federal University of Ceará (UFC) (number 38/15).

### Experimental periodontitis

For the induction of experimental periodontitis (EP) it was used the model of ligature-induced periodontitis (Goes et al., 2010). The animals were anesthetized with ketamine (70 mg/kg administered i.p., 10% Quetamina, Vetnil, São Paulo, SP, Brazil) and xylazine (10 mg/kg administered i.p., 2% Calmium, São Paulo, SP, Brazil). Later, a sterilized nylon (000) thread ligature was placed around the cervix of the second left upper molar. The ligature was then knotted on the vestibular side of the referred tooth. Eleven days after ligature placement the animals were euthanized with 20 mg/kg thiopental (0.5 g Thiopentax, Cristália, São Paulo, SP, Brazil). The ligatures were blinded to the group.

### Experimental groups

The animals (*n* = 6 in each group) were randomly assigned into 3 groups: SAL, CLO and Naïve. The rats received, according to the group, either 0.9% of Saline solution (SAL) at the dose of 2 ml/kg or 90 mg/kg of aqueous flower extract of *Calendula officinalis* (CLO) (Batch number PROD004257) purchased from Mapric Cosmetic and Pharmaceutical Products, Brazil (chemical abstract service—CAS – number 64-17-5; 7732-18-5; 99-76-3), by gavage, 30 min before EP and daily for 10 days. The Naïve group experienced no manipulation.

### Macroscopic analysis of alveolar bone

On the 11th day, animals were euthanized under anesthesia and had their maxillae removed and fixed in 10% neutral formalin for 24 h. Following, maxillae were divided in half, dissected and stained with 1% methylene blue (Goes et al., 2010). For the measurement of ABL, hemi-maxillae were placed in microscope slides and photographed with a digital camera (Nikon® D40, Melville, NY, USA). The acquired image was analyzed using the IMAGE J® software (ImageJ 1.32j; National Institute of Health, Bethesda, MD, USA), according to the methodology described by Goes et al. (2010).

### Scanning electron microscopy (SEM) of alveolar bone

Two additional groups of 6 animals submitted to EP, which received SAL or CLO (90 mg/kg), were euthanized and had their maxillae removed. The specimens were fixed in Karnovisky for at least 6 h, then they were held in a Cacodylate buffer. The maxillae were cut in a diamond blade cutter, in a mesial-distal plane, to obtain the maxilla fragment (0.5 × 0.2 cm and 0.5 mm thick). The fragment was placed in an eppendorf tube and left in the desiccator drying for 24 h. The fragments were assembled into stubs for metallization with gold powder (Quorum Metallizer QT150ES, Quorum Technologies, Laughton, England) for the analysis by a scanning electron microscopy (SEM inspect-50, FEI, Hillsboro, Oregon, USA). It was evaluated the bone topography of the interproximal region between the first and second maxillary left molars (Lu et al., 2014).

In addition, it was used the Energy Dispersive Spectroscopy (EDS), which is a detector (Oxford Instruments, Abingdon, Oxfordshire, UK) installed in the vacuum chamber of the MEV. The elemental and chemical analysis of the sample were performed using the manufacturer's software (AztecEnergy, Oxford Instruments, Abingdon, Oxfordshire, UK).

### Analysis of collagen fibers in periodontium

Two additional groups of 6 animals submitted to EP, which received SAL or CLO (90 mg/kg), were euthanized and had their maxillae removed. The specimens were fixed in 10% neutral buffered formalin for 24 h, and then demineralized in 10% EDTA for 30 days. Following this, the specimens were dehydrated, embedded in paraffin and sectioned (4 μm) along the molars in a mesiodistal plane for H&E staining (Goes et al., 2012). Considering that collagen is a structural protein that presents a natural phenomena of self-fluorescence, the images were obtained using the Confocal LSM 710 microscope (Zeiss, Jena, Germany) and analyzed by manufacture's software (Zen 2.1 lite black, 64-bit version, 758 MB, Zeiss, Jena, Germany). In order to evaluate the presence and organization of collagen fibers in periodontium, between the first and second left upper molar, it was used 488 nm wavelength laser and emission channel for FITC-green fluorescence (Gonçalves et al., 2014).

In order to identify the presence and type of fibrilar collagen, histological sections (4 μm) were obtained from the previously prepared paraffin blocks, and were stained with Picrosirius Red. The slides were evaluated under a polarized light filter. Collagen birefringence, as yellow-red for type I collagen and green for type III collagen (Junqueira et al., 1979), was evaluated between the first and second maxillary left molars.

### Immunohistochemical analyses of DKK1, WNT 10b and β-catenin

The streptavidin–biotin–peroxidase method was used for Immunohistochemistry assay, in paraffin-embedded tissue sections 4 μm thick. Sections of the excised maxillae, demineralized in a 10% EDTA solution were used for DKK1 (antibody rabbit policlonal IgG, Santa Cruz Biotechnology), WNT 10b (antibody rabbit policlonal IgG, Abcam), and β-catenin (antibody goat policlonal IgG, Santa Cruz Biotechnology) (Sousa et al., 2016). Five microscopic fields (400x) were used to count osteoblasts exhibiting cytoplasmic positivity for DKK1, WNT 10b and β-catenin (de Barros Silva et al., 2016).

### Gingival levels of reduced glutathione, enzyme catalase, enzyme superoxide dismutase and malondialdehyde

Reduced glutathione (GSH), catalase (CAT) superoxide dismutase (SOD) and malondialdehyde (MDA) were performed to evaluate oxidative stress. For this, the gingival tissue was removed 11 days after EP, then stored at −80°C. The level of GSH in gingival tissue was estimated according to the methods described previously (Sedlak and Lindsay, 1968). The GSH concentration was expressed as micrometers of GSH per gram of wet tissue.

Superoxide dismutase (SOD) activity was assayed as described previously (Beauchamp and Fridovich, 1971). In a dark room, the gingival samples were homogenized in 20 μl of ice-cold phosphate buffer at 15,000 G for 20 min. The supernatants were mixed with a solution comprised of phosphate buffer (50 nM), EDTA (100 nM) and L-methionine (19.5 mM) in a pH of 7.8. Then, 150 ml of a solution of riboflavin (10 nM) and NBT (750 nM) as added and the mixture was exposed to light (20 W) for 15 min. The absorbance of the samples was measured at 560 nm. The results are expressed as grams of SOD per ml.

CAT activity has as principle the measurement of O_2_ production rate and H_2_O in proportion of H_2_O_2_ (Maehly and Chance, 1954). Briefly, 20 μl of gingival homogenate was mixed with a solution comprised of 3% H_2_O_2_ and Tris-HCl EDTA buffer (5 nM, pH 8.0). The absorbance at a 230 nm wavelength was measured immediately and 6 min after preparing the samples.

Malondialdehyde (MDA) indicates lipid peroxidation based on the reaction of this substance with thiobarbituric acid, in the gingival tissue of rats. Briefly, 250 μl of 10% homogenate of gingival tissue were mixed with 1.5 ml of 1% H3PO4 and 0.5 ml of 0.6% thiobarbituric acid aqueous solution and the mixture was stirred and heated in boiling water for 45 min. After cooling, 2 ml of n-butanol were added and the mixture was homogenized. The butanol layer was separated, and the difference between the optical densities at 535 was used for calculating the MDA concentrations, which were expressed as nanomol of MDA per gram of gingival tissue (Mihara and Uchivam, 1978).

### Statistical analysis

Data are presented as mean ± SEM. In order to compare means it was used Analysis of variance (ANOVA) followed by Bonferroni test. *P* < 0.05 was set to indicate significant differences among groups. All analyses were performed using GraphPad Prism 6 software, San Diego, CA, USA.

## Results

### Effect of CLO on alveolar bone loss

**Figure 1:**
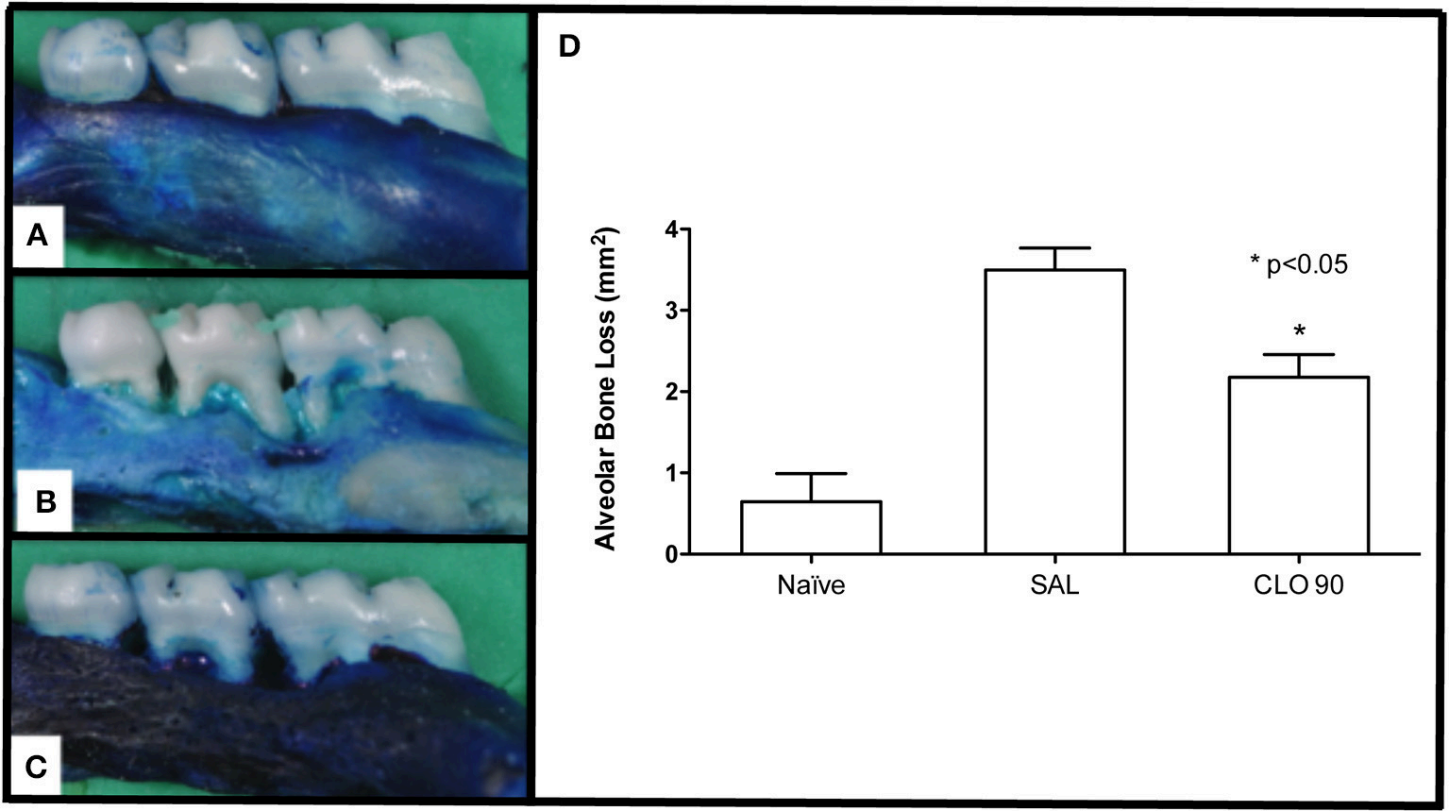
Effect of CLO on ABL in rats with EP. Naoïve hemimaxilla **(A)**, Hemimaxilla from SAL group **(B)**, Hemimaxilla tretated with CLO 90 mg/kg **(C)**, Macroscopic Analysis **(D)**. Bars represent the mean ± SEM of 6 animals per group. ^*^P < 0.05 was considered to be significantly different compared with SAL (ANOVA followed by the Bonferroni test). (**A–C**, 4x magnification).

Morphometric study demonstrated that the experimental periodontitis caused intense bone resorption (Figure 1D) compared to the normal hemi-maxillae from Naïve group (Figure 1A). Rats from SAL group presented intense alveolar resorption, root exposure and furcation lesion (Figure 1B). Ninety mg/kg of CLO prevented bone loss (Figure 1C), by 42.8%, when compared to SAL (*p* < 0.05). The treated animals showed greater preservation of bone tissue.

### Effect of CLO on bone topography and mineral distribution

**Figure 2:**
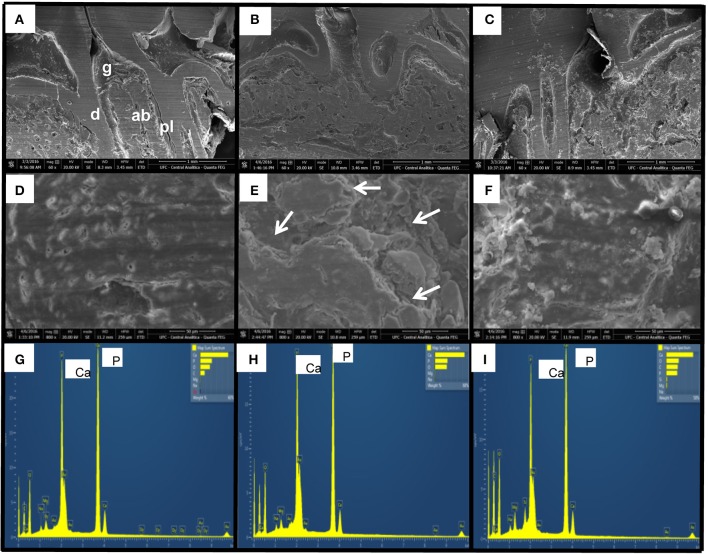
Effect of CLO on topography and mineral distribution of alveolar bone of rats with EP. Naïve **(A,D,G)**, SAL **(B,E,H)**, CLO **(C,F,I)**. Dentin (d); Alveolar bone (ab); Periodontal ligament (pl); Gingiva (g). Arrows indicate irregularity on bone tissue. (Magnification 60x **A–C**; Magnification 800x **D–F**).

Experimental Periodontitis (Figure 2B) caused important destruction of bone tissue when compared to the normal tissue from Naïve group (Figure 2A). CLO, at 90 mg/kg, prevented bone loss (Figure 2C). In 800x magnification, it was possible the observe that the bone tissue from an animal of SAL group (Figure 2E) presented an irregular topography, when compared to the bone tissue of an animal from Naïve group (Figure 2D). The treatment with CLO (Figure 2F) kept the tissue topography more regular than the one seen on SAL group.

The EDS analysis allowed the immediate identification of minerals and the chemical elements distribution on the samples (Figures 2G–I). It was possible to see elevated peaks of calcium and phosphorus on bone tissue, without any difference between groups.

### Effect of CLO on collagen fiber of periodontium

**Figure 3:**
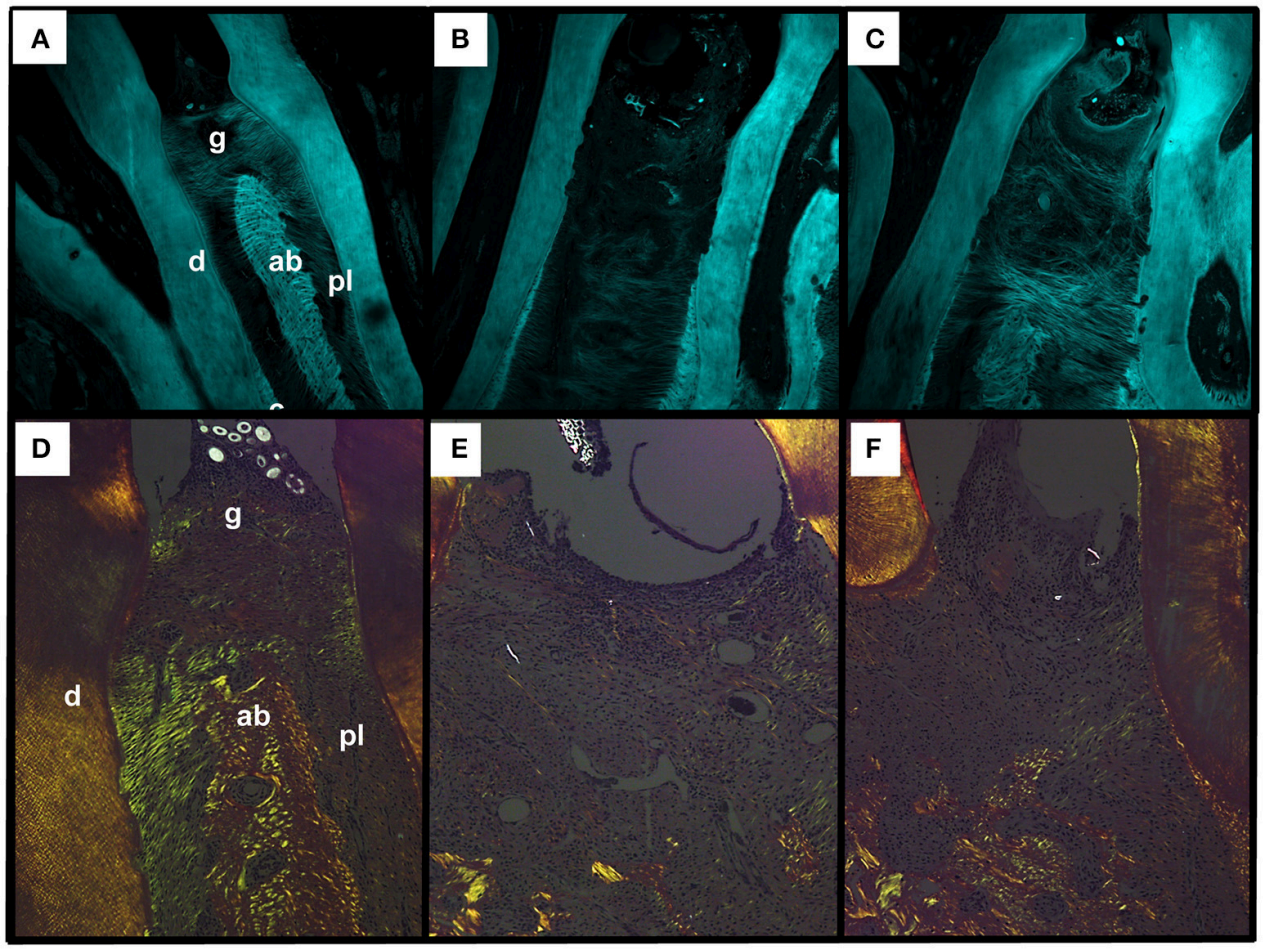
Effect of CLO on collagen fibers of alveolar bone of rats with EP. Naïve **(A,D)**, SAL **(B,E)**, CLO **(C,F)**. Dentin (d); Alveolar bone (ab); Periodontal ligament (pl); Gingiva (g). (Magnification 40x).

The confocal analysis of periodontium of animals from SAL group (Figure 3B) demonstrated great destruction and derangement of collagen fibers in periodontal ligament compared to the Naïve group (Figure 3A). The treatment with 90 mg/kg CLO (Figure 3C) preserved these collagen fibers.

Using picrosirius red staining under polarized light filter, it was possible to observe that the majority of collagen fibers from periodontal ligament from animals of Naïve group showed green color (Figure 3E) The collagen fiber of periodontium of animals from SAL group presented reduction on the birefringencewhen compared to Naïve (Figure 3D) The treatment with CLO caused a slight increase on birefringence when compared to SAL, showing fibers in greenish color (Figure 3F).

### Effect of CLO on immunolabeling of WNT10b, DKK-1 and beta-catenin

**Figure 4:**
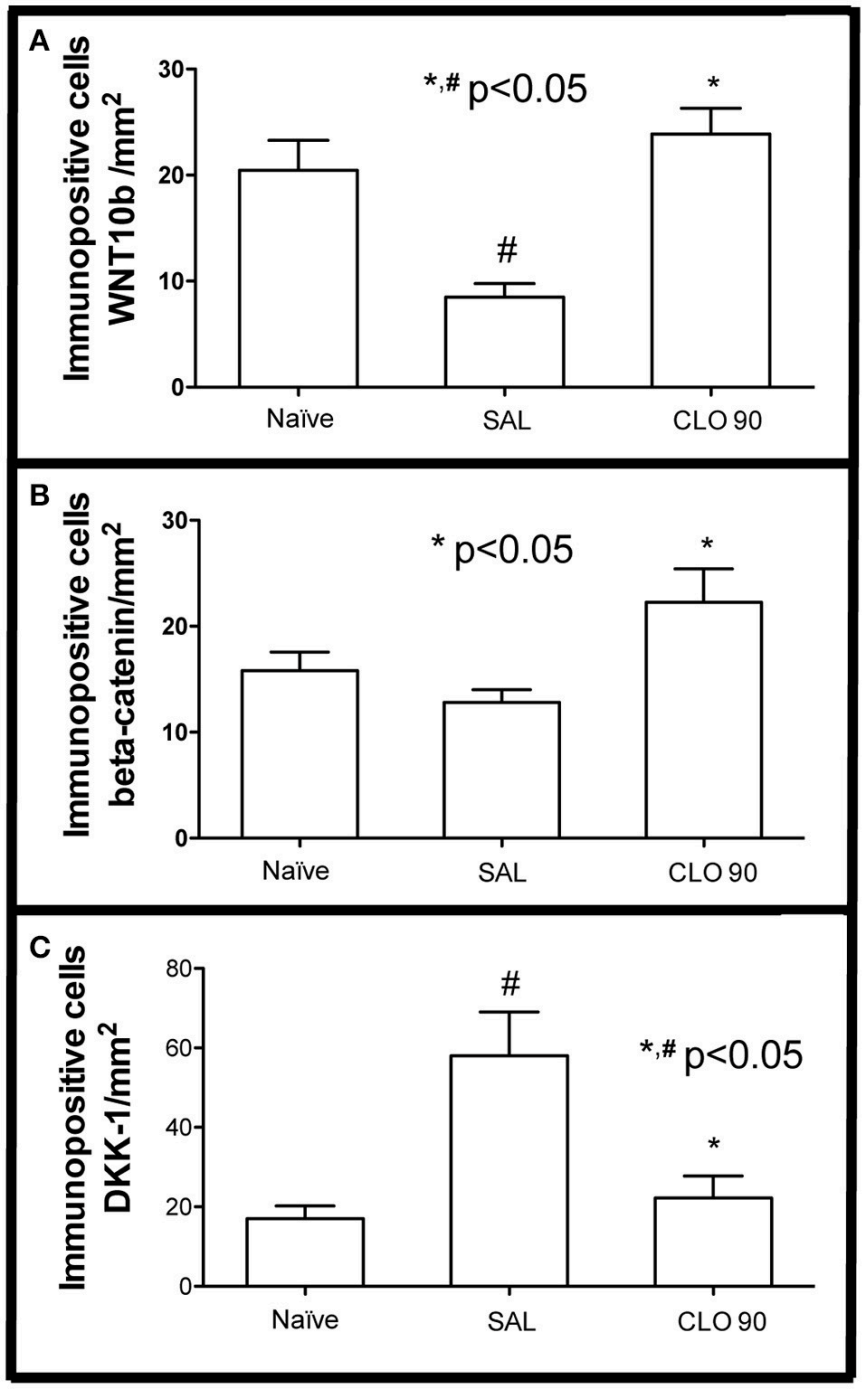
Effect of CLO on quantification of immunopositive cells for markers of WNT pathway. WNT 10b **(A)**, beta-catenin **(B)**, DKK-1 **(C)**. Bars represent the mean ± SEM of 6 animals per group. ^#^*P* < 0.05 was considered to be significantly different compared with Naïve. ^*^P < 0.05 was considered to be significantly different compared with SAL (ANOVA followed by the Bonferroni test).

**Figure 5:**
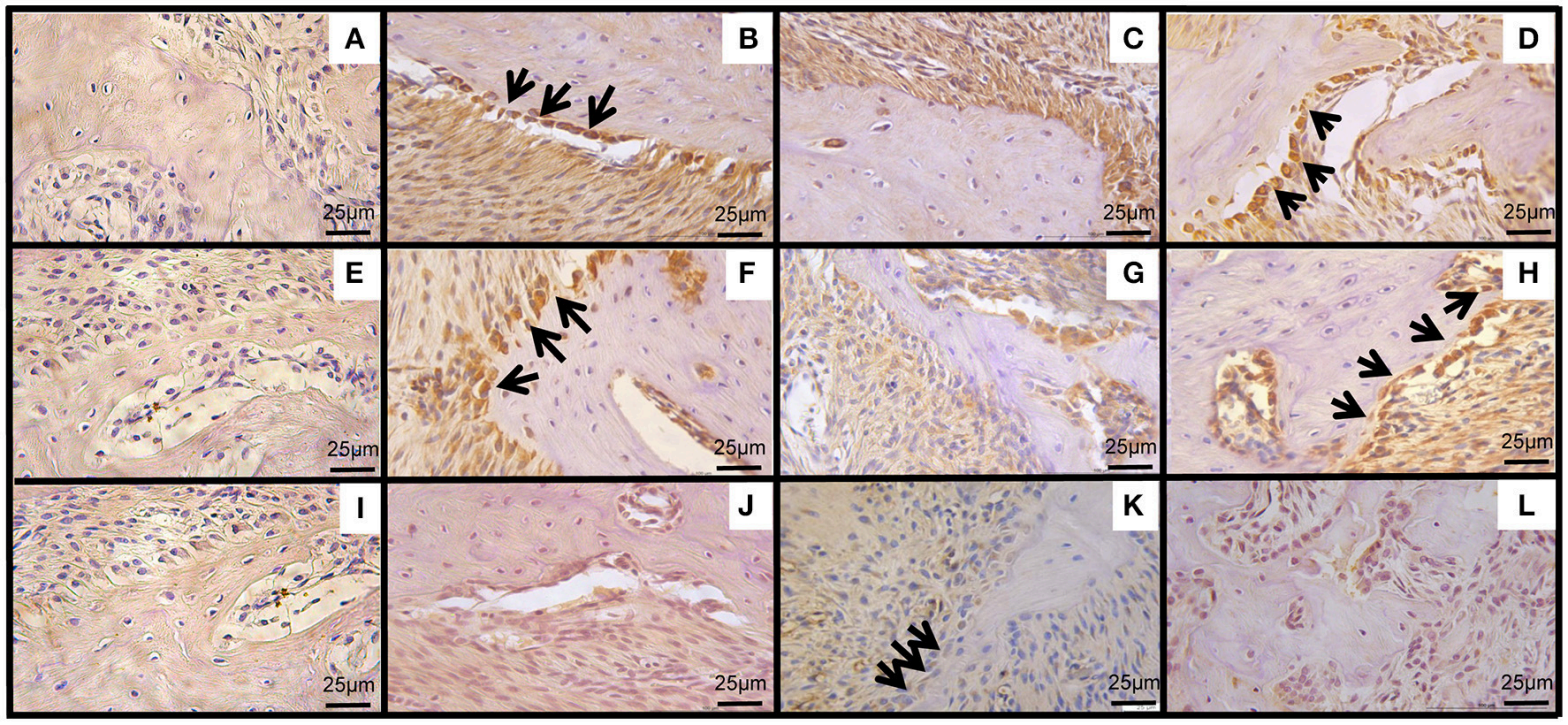
Effect of CLO on immunoexpression of WNT pathway in periodontium of rats with. WNT10b **(B–D)**, β-catenin **(F–H)** and DKK-1 **(J–L)**, between first and second molar of a periodontium from Naïve group **(B,F,J)**, periodontium from SAL group **(C,G,K)**, periodontium of animals treated with CLO 90 mg/kg **(D,H,L)**. Negative controls of WNT10b **(A)**, β-catenin **(E)**, DKK-1 **(I)**. (Magnification 400x). (→) indicate immunopositive osteoblasts. Bar = 25 μm.

Considering the canonical WNT pathway, the animals submitted to EP that received SAL showed reduction on the amount of immunopositive cells to WNT 10b (8.50 ± 1.25 positive cells/mm^2^) (Figures 4A, 5C) (*p* < 0.05), β-catenin (12.80 ± 1.20) (Figures 4B, 5G) (*p* > 0.05), and an increase to DKK-1 (58.00 ± 11.03) (Figures 4C, 5K) (*p* < 0.05) when compared to Naïve [WNT 10b = 22.44 ± 2.82 (Figure 5B); β–catenin = 15.80 ± 1.77 (Figure 5F); DKK-1 = 17.00 ± 3.21 (Figure 5J)]. The treatment with CLO 90 mg/kg increased the count of immunopositive cells for WNT 10b (23.89 ± 2.41) (Figure 5D) and β-catenin (22.25 ± 3.16) (Figure 5H) (*P* < 0.05) when compared to SAL (WNT 10b = 8.50 ± 1.25 and β-catenin = 12.80 ± 1.20). There was a significant decrease on the count of immunopositive cells for DKK-1 in CLO group (22.20 ± 5.55) (Figure 5L), compared to SAL (58.00 ± 11.02). The negative controls for WNT 10b, β-catenin, and DKK-1 can be seen on Figures 5A,E,I, respectively.

### Effect of CLO on gingival GSH, SOD, CAT, and MDA levels

**Figure 6:**
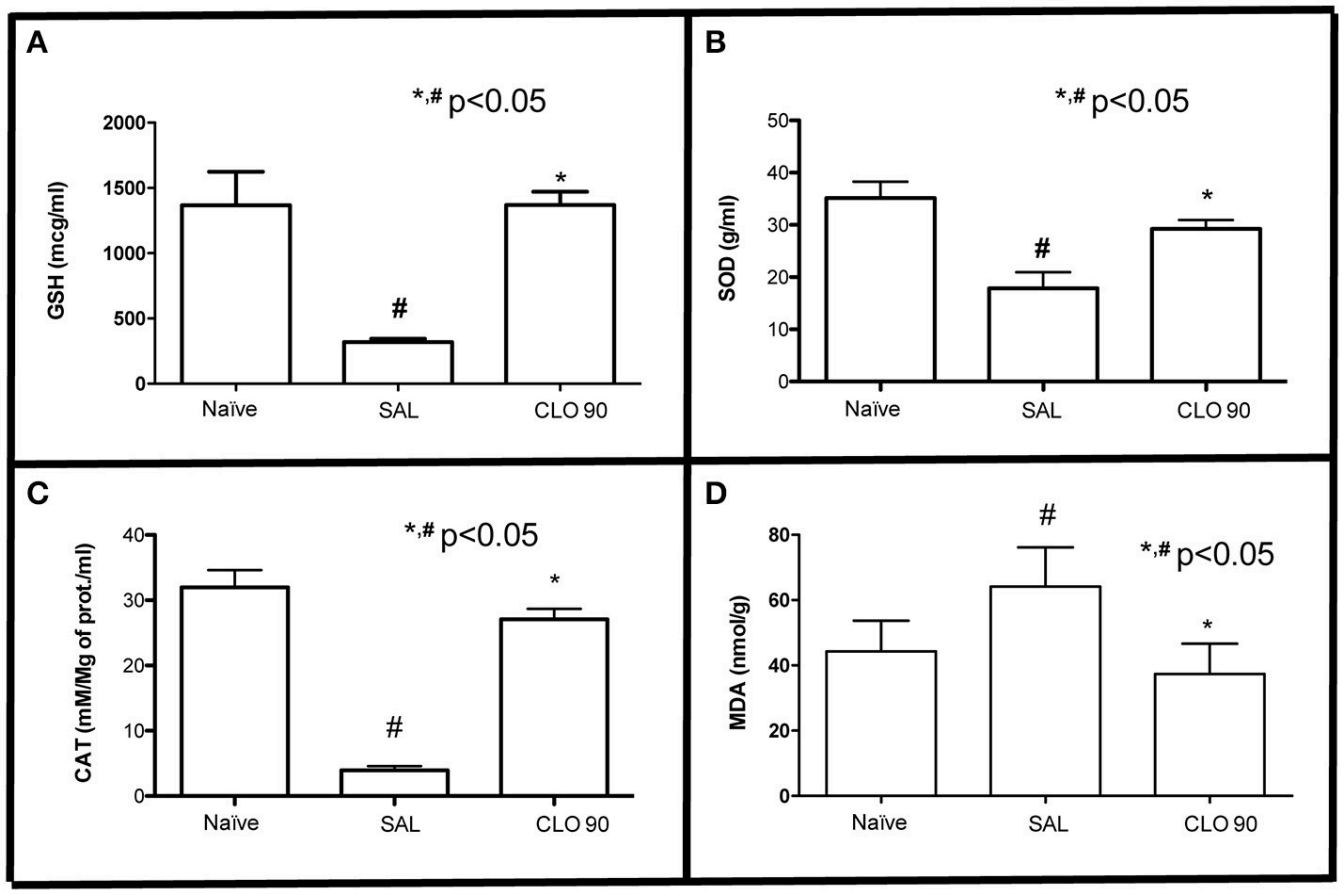
Effect of CLO on oxidative stress markers in gingival tissue of rats with EP. GSH **(A)**, SOD **(B)**, CAT **(C)**, MDA **(D)**. Bars represent the mean ± SEM. ^#^*P* < 0.05 was considered to be significantly different compared with Naïve. ^*^P < 0.05 was considered to be significantly different compared with SAL (ANOVA followed by the Bonferroni test).

The experimental periodontitis caused significant reduction of the GSH (Figure 6A), SOD (Figure 6B) and CAT (Figure 6C), as well as an increase on MDA (Figure 6D) gingival levels (*P* < 0.05) when compared to the Naïve group, suggesting that oxidative stress is observed in gingival tissue subjected to periodontitis. Administration of 90 mg/kg CLO increased gingival GSH, SOD and CAT concentration, and reduced MDA levels compared with SAL group.

## Discussion

In order to study periodontitis, it has been well described on literature the use of animal models, among them the one of ligature-induced model in rats. Several studies have shown that this model is able to reproduce the main characteristics seen on human periodontitis, such as bone loss and destruction of periodontal ligament (Goes et al., 2010, 2016; Sousa et al., 2016) increase on oxidative stress (Di Paola et al., 2004), and exacerbation of inflammatory process (Leitão et al., 2005; Goes et al., 2012, 2016; Sousa et al., 2016), which confirm our findings.

Therefore, considering the important role of inflammation and oxidative stress on bone resorption, it seems interesting the use of pharmacological agents that present anti-inflammatory and antioxidant actions showing low incidence of adverse effects and of low-cost, as observed in several natural products. So *Calendula officinalis* (CLO) stands out as a modulator of inflammation that can be used as adjuvant to the treatment of periodontitis.

On this study it was seen that CLO presented antirresorptive effect preventing bone loss and preserving its topography. Despite the lack of studies evaluating the effect of the crude extract of CLO on bone tissue, it has been reported that queretin, the main flavonoid found on CLO extract, is the one responsible, in great part, of the pharmacological effects of this plant (Saini et al., 2012). *In vitro*, quercetin significantly increased osteoblast differentiation (Zhou et al., 2015) and induced mRNA expression of sialoprotein and osteocalcin in osteoblast culture (Satué et al., 2013). *In vivo*, quercetin inhibited bone loss in periodontitis models in rats (Napimoga et al., 2013), increased serum osteocalcin and the activity of alkaline phosphatase (Liang et al., 2011), contributing to bone tissue preservation.

By occasion of SEM analysis, we performed the EDS evaluation that can analyze the chemical elements compounds, showing its distribution on the sample surface (Newbury and Ritchie, 2015). Our results showed no difference on the chemical compounds of bone tissue considering the experimental groups, what indicates that CLO does not affect the type and/or amount of minerals when compared to the normal tissue.

Considering WNT pathway, our results showed, for the first time, the effect of CLO on this signaling pathway, by the increase of WNT10b and beta-catenin, while it reduced DKK-1 immunoexpressions. Among the several types of well-known WNT proteins in mammals (Kikuchi, 2009) WNT10b stands out as a positive modulator of bone formation (Stevens et al., 2010). This pathway is regulated by DKK and Sclerostin (SOST), two extracellular antagonists, which have their expression increased during inflammatory conditions (Wang et al., 2011). So the effect of CLO on WNT pathway may be related to the anti-inflammatory effect of this plant (Preethi et al., 2009).

In addition, it has been described a relationship between oxidative stress and WNT pathway. WNT proteins activate the Frizzled/LRP5 or LRP6 receptor complex preventing β-catenin degradation (Clevers and Nusse, 2012), allowing its association with the T cell factor (TCF) lymphoid-enhancer binding factor (LEF) family of transcription factors which regulates the expression of WNT-target genes, such as Osterix1 in bone-producing osteoblasts (Rodda and McMahon, 2006). However, during stress conditions, the high levels of reactive oxygen species (ROS) promotes the activation of the transcription fator, Forkhead box O (FOXO) (Iyer et al., 2013). The binding of β-catenin to FOXOs diverts β-catenin from Wnt/TCF- to FOXO-mediated transcription decreasing osteoblastogenesis *in vitro* (Almeida et al., 2007). These findings highlight the beneficial anti-inflammatory effect of CLO on bone metabolism.

This study performed analyses of collagen fibers. CLO preserved collagen confirming others studies which already showed that CLO extract reduced collagen breakdown (Millán et al., 2016), and increased collagen concentration (Aro et al., 2015). In gingival fibroblasts, CLO inhibited matrix metalloproteinases (MMP)-2 (Saini et al., 2012). Collagen is the main constituent of periodontal ligament and plays a key role in the architecture of the periodontium (Kaku and Yamauchi, 2014). Therefore, collagen breakdown is understood as the main marker of periodontal disease progression (de Almeida et al., 2015). Moreover, evaluating its birefringence, in CLO group the collagen fibers were, in its majority, green (Type III collagen). Type III collagen is observed on the initial phases of healing (Li and Sae-Lim, 2007), and it is considered essential to further production of type I collagen (Liu et al., 1997).

The oxidative stress is a characteristic of inflammatory process, and it is considered an important factor on periodontal pathogenesis (Chapple and Matthews, 2007). CLO reduced the periodontal oxidative stress, reestablishing GSH, SOD and CAT, important antioxidant enzymes, and reducing MDA levels on periodontal tissue, a marker of tissue destruction, as in accordance to other studies (Tanideh et al., 2016; Verma et al., 2016). Quercetin seems to play a role on this effect, since it has exhibited antioxidant activity (Fonseca et al., 2010). According to Heijnen et al. (2002) this effect is attributed to the presence of two antioxidant pharmacophores within the molecule of quercetin that have the optimal configuration for free radical scavenging.

In summary the present findings showed that CLO exhibited antiresorptive effect, preserved collagen fibers and presented antioxidant activity with participation of WNT signaling pathway. Therefore, this natural product deserves further investigation as pharmacological tool for preventing periodontal bone loss.

## Author contributions

ML and AL induced periodontitis, treated the animals, performed all the assays. CM performed immunohistochemical assay, GB performed microscopy analysis. VC performed SEM assay and analysis, PG was the supervisor of the study.

### Conflict of interest statement

The authors declare that the research was conducted in the absence of any commercial or financial relationships that could be construed as a potential conflict of interest.
